# Whole-Genome Sequence of *Leptospira interrogans* Serovar Hardjo Subtype Hardjoprajitno Strain Norma, Isolated from Cattle in a Leptospirosis Outbreak in Brazil

**DOI:** 10.1128/genomeA.01302-15

**Published:** 2015-11-05

**Authors:** M. R. V. Cosate, S. C. Soares, T. A. Mendes, R. T. Raittz, E. C. Moreira, R. Leite, G. R. Fernandes, J. P. A. Haddad, J. Miguel Ortega

**Affiliations:** a Centro de Biotecnologia, Instituto Butantan, São Paulo, São Paulo, Brazil; b Instituto de Ciências Biológicas e Naturais, Universidade Federal do Triângulo Mineiro, Uberaba, Minas Gerais, Brazil; c Departamento de Bioquímica e Imunologia, Instituto de Ciências Biológicas, Universidade Federal de Minas Gerais, Belo Horizonte, Minas Gerais, Brazil; d Laboratório de Bioinformática, Setor de Educação Profissional e Tecnológica, Universidade Federal do Paraná, Curitiba, Paraná, Brazil; e Departamento de Medicina Veterinária Preventiva, Escola de Veterinária, Universidade Federal de Minas Gerais, Belo Horizonte, Minas Gerais, Brazil; f Genômica e Biologia Computacional, Centro de Pesquisas René Rachou, Fiocruz, Belo Horizonte, Minas Gerais, Brazil

## Abstract

Leptospirosis is caused by pathogenic bacteria of the genus *Leptospira* spp. This neglected re-emergent disease has global distribution and relevance in veterinary production. Here, we report the whole-genome sequence and annotation of *Leptospira interrogans* serovar Hardjo subtype Hardjoprajitno strain Norma, isolated from cattle in a livestock leptospirosis outbreak in Brazil.

## GENOME ANNOUNCEMENT

Leptospirosis is a global disease caused by spirochetes belonging to the genus *Leptospira* spp., which comprise both saprophytic and pathogenic species (1). Taxonomic classification of the genus includes eight pathogenic species of *Leptospira*, and over 200 pathogenic serovars (2, 3). Leptospirosis remains recognized as an important zoonotic disease that affects humans and animals (4, 5). In cattle herds, the *L. interrogans* serovar Hardjo subtype Hardjoprajitno is directly associated with clinical conditions such as reproductive failure, including abortion, mastitis, and a reduction in milk production (6, 7), while *L. borgpetersenii* serovar Hardjo subtype Hardjobovis was reported as being more adapted to the reservoir host. The bacterial genome presented here was isolated from cattle with clinical signs of leptospirosis in livestock production. The bacteria was cultured for 4 months with routine inspection and was initially identified by serological and molecular approaches using 16S rRNA and *secY* genes, confirming this bacterial strain as a member of the genera *Leptospira* spp. and *L. interrogans* spp. (7).

The DNA from *L. interrogans* serovar Hardjo subtype Hardjoprajitno strain Norma (hereafter LIHH_Norma) was obtained from lysed cells using lysozyme solution, and the DNA was extracted using phenol/chloroform/isoamyl alcohol (8). Library construction, titration, emulsion PCR, and sequencing steps were performed according to the manufacturer’s protocol. The DNA was sequenced using the 454 GS-FLX Titanium platform with two libraries: shotgun, resulting in 599,359 single reads with a coverage of ~76×; and, pair-end, resulting in 879,022 reads with a coverage of ~40× constructed using a 3-Kb-long tag (9).

A total of 21,692,784 bp and 39,835,204 bp were assembled into 7 scaffolds with a sequence coverage of ~50.2× using Newbler version 2.6, and the resulting gaps were closed using FGAP (10). For the overlap detection, we configured the seed step with the parameter “8,” and the seed length with “16.” The minimum overlap length considered was 40 nucleotides with a minimum identity of 90 percent.

Automatic transfer of genome annotation was performed using “in-house” scripts and *L. interrogans* serovar Lai strain 56601 as reference. Also, automatic annotation was performed using RAST version 2.0, and additional genes were manually included into the genome of LIHH_Norma using Artemis version 16.0.0 (11, 12). Finally, tRNAs and rRNAs were predicted using tRNAscan-SE version 1.21 and RNAmmer version 1.2, respectively (13, 14). The predicted 16S rRNA from RNAmmer was queried using BLASTn against the NCBI NT database to reconstruct the phylogenetic tree of the genus and confirm the taxonomic classification of the strain.

The genome sequence of LIHH_Norma consists of two circular chromosomes, 4,406,718 bp and 355,432 bp in length with ~35.02% and ~35.04% G+C content, respectively. Chromosome I has 4,495 genes, composed of 4,348 coding sequences (CDSs), 37 tRNAs, 5 rRNAs (1 5S, 2 16S, and 2 23S) and 105 pseudogenes, whereas chromosome II has 355 genes, composed of 348 CDSs and 7 pseudogenes. The obtained genome sequence is highly important for future epidemiological studies of *Leptospira* spp. isolated in Brazil.

### Nucleotide sequence accession numbers.

The annotated assembly is available in GenBank under the accession numbers CP012603 (CI) and CP012604 (CII), Bioproject PRJNA 185511, and Biosample SAMN 03853357.
